# Simultaneous [^18^F]FDG-PET/MRI: Correlation of Apparent Diffusion Coefficient (ADC) and Standardized Uptake Value (SUV) in Primary and Recurrent Cervical Cancer

**DOI:** 10.1371/journal.pone.0141684

**Published:** 2015-11-09

**Authors:** P. Brandmaier, S. Purz, K. Bremicker, M. Höckel, H. Barthel, R. Kluge, T. Kahn, O. Sabri, P. Stumpp

**Affiliations:** 1 Department of Diagnostic and Interventional Radiology, University Hospital Leipzig, Leipzig, Germany; 2 Department of Nuclear Medicine, University Hospital Leipzig, Leipzig, Germany; 3 Department of Gynecology and Obstetrics, University Hospital Leipzig, Leipzig, Germany; The Norwegian University of Science and Technology (NTNU), NORWAY

## Abstract

**Objectives:**

Previous non–simultaneous PET/MR studies have shown heterogeneous results about the correlation between standardized uptake values (SUVs) and apparent diffusion coefficients (ADCs). The aim of this study was to investigate correlations in patients with primary and recurrent tumors using a simultaneous PET/MRI system which could lead to a better understanding of tumor biology and might play a role in early response assessment.

**Methods:**

We included 31 patients with histologically confirmed primary (n = 14) or recurrent cervical cancer (n = 17) who underwent simultaneous whole-body ^18^F-FDG-PET/MRI comprising DWI. Image analysis was performed by a radiologist and a nuclear physician who identified tumor margins and quantified ADC and SUV. Pearson correlations were calculated to investigate the association between ADC and SUV.

**Results:**

92 lesions were detected. We found a significant inverse correlation between SUV_max_ and ADC_min_ (r = -0.532, *p = 0*.*05*) in primary tumors as well as in primary metastases (r = -0.362, *p = 0*.*05*) and between SUV_mean_ and ADC_min_ (r = -0.403, *p = 0*.*03*). In recurrent local tumors we found correlations for SUV_max_ and ADC_min_ (r = -0.747, *p = 0*.*002*) and SUV_mean_ and ADC_min_ (r = -0.773, *p = 0*.*001*). Associations for recurrent metastases were not significant (p>0.05).

**Conclusions:**

Our study demonstrates the feasibility of fast and reliable measurement of SUV and ADC with simultaneous PET/MRI. In patients with cervical cancer we found significant inverse correlations for SUV and ADC which could play a major role for further tumor characterization and therapy decisions.

**Key Point 1**

This study investigates the correlation of functional parameters in a simultaneous PET/MRI.

**Key Point 2**

We found significant inverse correlations between ADC and SUV in cervical carcinoma which could increase knowledge about tumor biology.

## Introduction

According to global cancer statistics, cervical carcinoma is one of the most frequent cancers diagnosed in women [1]. Thorough clinical investigations and a precise initial staging are mandatory to assess local tumor extension and possible lymph node infiltration. Currently, sophisticated routine clinical imaging to stage cervical cancer patients is primarily performed with high resolution pelvic magnetic resonance imaging (MRI). According to the European Society for Medical Oncology (ESMO) guidelines [2], positron emission tomography (PET) is an adequate tool with high sensitivity and specificity to accurately delineate the extent of disease, especially in lymph nodes that are not macroscopically enlarged and in distant sites. Both PET and MRI provide functional parameters: standardized uptake value (SUV) for glucose metabolism from PET and apparent diffusion coefficient (ADC) measured with diffusion weighted imaging (DWI) for Brownian motion of water molecules in MRI. These measurements and the quantificiation of their correlation could add valuable information about tumor biology, which may contribute to a more sophisticated tumor characterization and finally could lead to a “tailored” therapy approach by estimating response behavior.

Measurement of SUV is commonly used as a semi-quantitative read-out of ^18^F-FDG uptake to supplement visual interpretation of the PET images. For example it is described to be correlated with histopathological grade, tumor cellularity and proliferative activity in sarcomas [3]. Regarding cervical cancer correlation with tumor aggressiveness and prognosis is reported in literature [4,5].

Nowadays DWI in MRI serves as a promising tool for the assessment and analysis of cellularity and discrimination of therapy responders in a variety of malignant tumor entities [6,7,8]

Depending on the degree of differentiation, a statistically significant difference between the ADCs of well-/moderately differentiated (G1/2) tumors and poorly differentiated (G3) tumors has been described in previous works [9, 10].

However, there is an ongoing controversy about the correlation of the functional parameters SUV and ADC. This is as some groups reported an inverse correlation, e.g. in head and neck squamous cell carcinoma [11]. Other data, e.g. from breast cancer or head and neck cancer patients, describe ADC and SUV as independent biomarkers [12,13] in non-simultaneous imaging environments. For cervical cancer, initial integrated PET/MR imaging results in 19 patients recently published by Grueneisen et al. revealed a significant and strong inverse correlation between SUV and ADC in primary tumors and associated primary lymph node metastases, but not in recurrent tumor lesions [14].

As compared to PET/CT simultaneous PET/MRI offers a new quality of hybrid cancer imaging by providing metabolic and high-resolution anatomic imaging with excellent soft tissue contrast. The aim of this study was to analyze a possible correlation of SUVs and ADC values derived from simultaneous PET/MRI in a larger and as such more representative population of patients with primary and recurrent tumors of the cervix uteri.

## Materials and Methods

This prospective study was approved by the local ethics committee (Ethic Committee of the Medical Faculty, University of Leipzig, Käthe—Kollwitz—Street 82, 04109 Leipzig, Germany) and all patients gave written informed consent.

### a) Patients

A total of 31 patients (range: 33–78y, mean ± sd: 55±13.7y) with primary (n = 14), or recurrent (n = 17) cervical cancer were examined employing a whole-body PET/MRI protocol over a time period of two years. Prior to the PET/MRI, all patients underwent a thorough clinical investigation by an experienced gynecologist. All tumors were histologically classified via tumor biopsy or intraoperative sampling (squamos cell carcinoma n = 25; adeno carcinomas n = 4; neuroendocrine tumors n = 2).

### b) Whole-body PET/MRI

The simultaneous PET/MRI system (Magnetom Biograph mMR—Biograph, Siemens Healthcare Sector, Erlangen, Germany) used in this study comprises a 3T whole-body scanner and a PET scanner.

The whole body simultaneous PET/MRI scan was performed from the skull to the upper thigh with 5 minutes per bed position (head, neck, chest, abdomen, pelvis, upper thighs) with simultaneous image acquisition. PET images were reconstructed using the iterative ordered subset expectation maximization algorithm with 3 iterations and 21 subsets, a Gaussian filter with 4 mm full width at half maximum (FWHM), and a 256 x 256 image matrix. Attenuation correction of the PET data was performed using a four-tissue (fat, soft tissue, air, background) model attenuation map which was generated from a Dixon-Vibe MR sequence [15].

Image acquisition started on average 130 minutes after intravenous administration of a body weight-adapted dose of ^18^F-FDG (4 MBq/kg, 192–442 MBq, mean±sd: 309±70.32 MBq) after a fasting period of at least 6 hours.

For whole-body MRI the following especially designed coils for PET/MRI were used: A spine coil and four body array coils were placed on the patient from the knee to the chest, the bed positions for head and neck imaging were covered using a dedicated head/neck coil. For procedural planning gradient-echo (GRE) localizer scans were used. In each PET bed position, the following four MR sequences were measured consecutively in free breathing (respiratory triggering was only used for the sequences in the abdominal bed position):

1. **T1w 3D GRE (Dixon-VIBE).** This obligatory sequence is used for attenuation correction of the PET images (coronal, TR 3.6 ms, TE 1.23ms, flip angle 10°, 128 slices, Slice thickness 2.6mm, FoV 500 x 500, voxel size 4.1 x 2.6 x 2.6mm, acquisition time 0:19 min/bed position.)
2. **T2w single-shot fast spin echo (HASTE).** For delineation of anatomy and gross pathology (transversal, TR 800 ms, TE 89 ms, flip angle 120°, 40 slices, Slice thickness 4mm, FoV 450 x 450, voxel size 1.8 x 1.4 x 4.0 mm, acquisition time 0:32 min/bed position).
3. **Diffusion-weighted echoplanar imaging (EPI-DWI).** For detection of restricted diffusion (monopolar sequence). B-values of 0 and 800 mm/s² were used with diffusion-sensitizing gradients applied in all three orthogonal directions (transversal, TR 6800 ms, TE 73 ms, 30 slices, Slice thickness 6mm, FoV 450 x 450, voxel size 3.1 x 1.6 x 5.0 mm, acquisition time 1:15 min/bed position).
4. **T2w-fat suppressed inversion recovery (TIRM).** As current standard in whole-body MR-based anatomy imaging (coronal, TR 2090 ms, TE 47 ms, flip angle 120°, 40 slices, Slice thickness 5mm, FoV 500 x 500, voxel size 4.3 x 3.5 x 6.0mm, acquisition time 1:00 min/bed position).

With the sequences described, a combined simultaneous whole-body PET/MR scan was realized within an average of 30 minutes examination time. Additionally we performed dedicated high—resolution pelvic MR-sequences to properly evaluate anatomical details and local tumor spread. However, these sequences were not used for image analysis or quantification of SUV or ADC.

### c) Image Analysis

**Fig 1:**
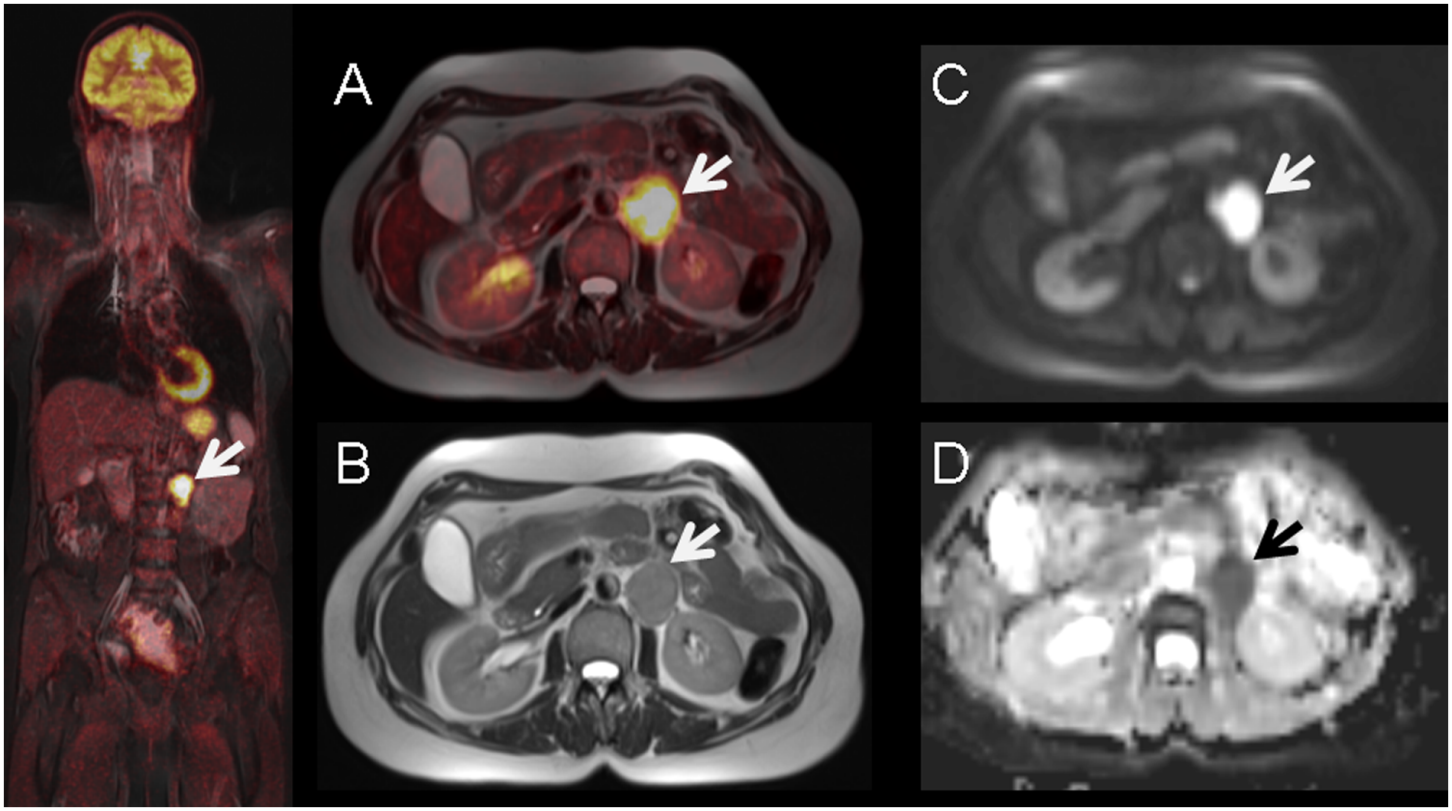
56-year old female with histology proven recurrent lymph node metastasis of cervical cancer diagnosed 3 years before. Pre-operative simultaneous ^18^F-FDG-PET/MRI (A) and T2-weighted MR imaging (B) show a hypermetabolic left paraaortal metastastic lymph node (arrow) with corresponding diffusion-restriction in DWI (C) and ADC-map (D).

An experienced radiological (6 years experience in gynaecological imaging) and nuclear medicine reader (7 years experience in oncological image interpretation) analyzed the images using dedicated viewing software (Syngo.via^®^; Siemens, Healthcare Sector, Erlangen, Germany). Lesions with focal uptake greater than the surrounding tissue (based on visual qualitative analysis) were considered as suspicious for malignancy (Fig 1).

**Fig 2:**
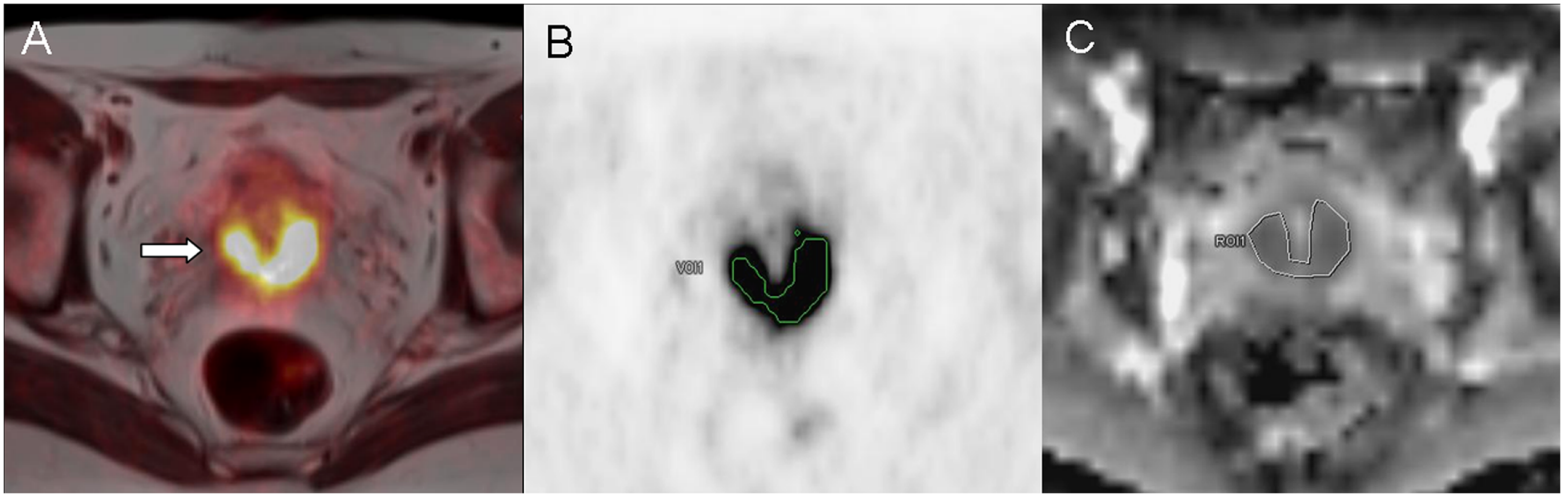
Lesion detection and definition of tumor margins (arrow) of primary cervical cancer in fused PET/MRI T2 –HASTE–sequence (A). Manual placement of a polygonal VOI in attenuation corrected PET images (B). ROI adjustment by manual segmentation on (C) the corresponding MRI ADC—map.

In 22 patients we could indentify multiple tumor-suspicious lesions. However, we only included tumor lesions, which could be detected in both modalities (SUV/ADC). Lesions that could only be detected in one imaging modality or were too small for reliable ADC measurements (<5mm) were not explicitely documented, so statements concerning interobserver agreement cannot be made. Detection of suspicious lymph nodes was mainly conducted via imaging (MR. PET-CT or PET/MR) according to FDG-uptake, size (>10mm), round shape or visible necrosis. To determine SUV_max_ and SUV_mean_, margins of tumor lesions were identified in MR images (T2 –sequence) and a volume of interest (VOI) was placed in the attenuation corrected PET dataset around the tumour (SUVmax threshold 40%). Region of interests (ROI) were then manually placed one each slice in the corresponding ADC map by the radiological reader (Fig 2). In order to ensure proper positioning of the ROI, identification of the tumor lesion was first conducted in a fused PET/T2w–Haste sequence, defining target lesions with high FDG uptake. However, analyzing small lesions (<10mm) was conducted via manual adjustment of polygonal ROIs, as the volume based method could not be applied sufficiently here. In some cases, however, minor manual adjustments (due to subtle misregistrations between PET and ADC) needed to be performed to guarantee an optimal ROI placement for the ADC measurement. As previously described in the literature, the main factors for misalignment in echo-planar imaging sequences are eddy current-induced image distortion and nonlinearities of the gradient coils [16].

To investigate a potential correlation between the SUV and ADC values, ADC maps were generated by the scanner software (syngo.via, Siemens, Erlangen, Germany) using two b—values (b = 0 s/mm2, b = 800 s/mm2). The following parameters were defined: ADC_mean_ as average ADC value for all voxels in each analyzed lesion and minimum ADC (ADC_min_) as the lowest ADC value among all. For PET maximum SUV (SUV_max_) and mean SUV (SUV_mean_) were recorded for all tumor lesions.

### d) Statistics

Statistical analysis was performed using IBM SPSS 20^™^ (SPSS Inc., Chicago, IL, USA). Data are presented as mean +/- standard deviation (SD). Descriptive analysis was used for SUV_max_, SUV_mean_, ADC_mean_ and ADC_min_ of primary or recurrent tumor tissue and/or lymph nodes/metastasis separately. To estimate a correlation between ADC and SUV correlation pairs were analyzed using Pearson’s correlation test. According to the classification system provided by Salkin, r values between 0.8–1.0 represent a very strong correlation, between 0.6 and 0.8 a strong correlation, between 0.4 and 0.6 a moderate correlation and between 0.2 and 0.4 a weak correlation. Values between 0.0 and 0.2 are classified as showing a weak or no relationship [17]. P values *≤* 0.05 were considered as statistically significant.

## Results

**Fig 3:**
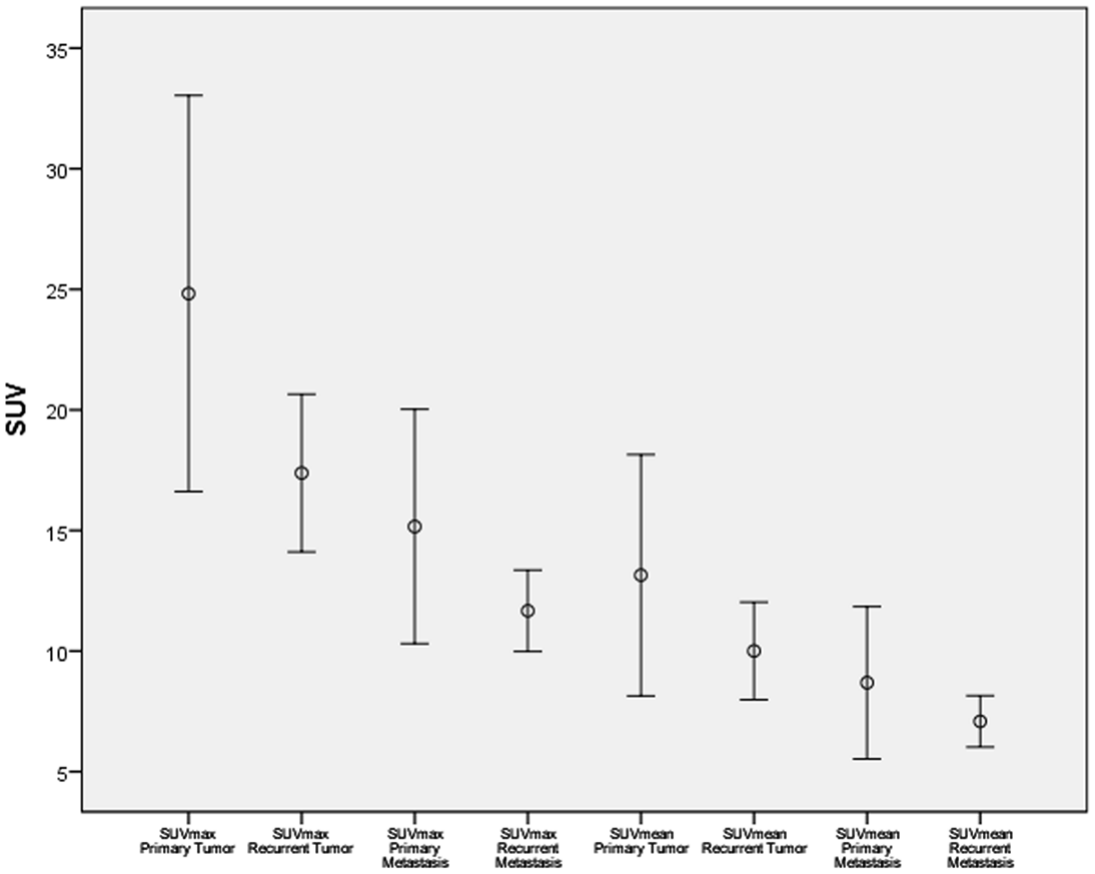
Absolute SUV of primary and recurrent cervical cancer/metastasis presented through error bars.

**Fig 4:**
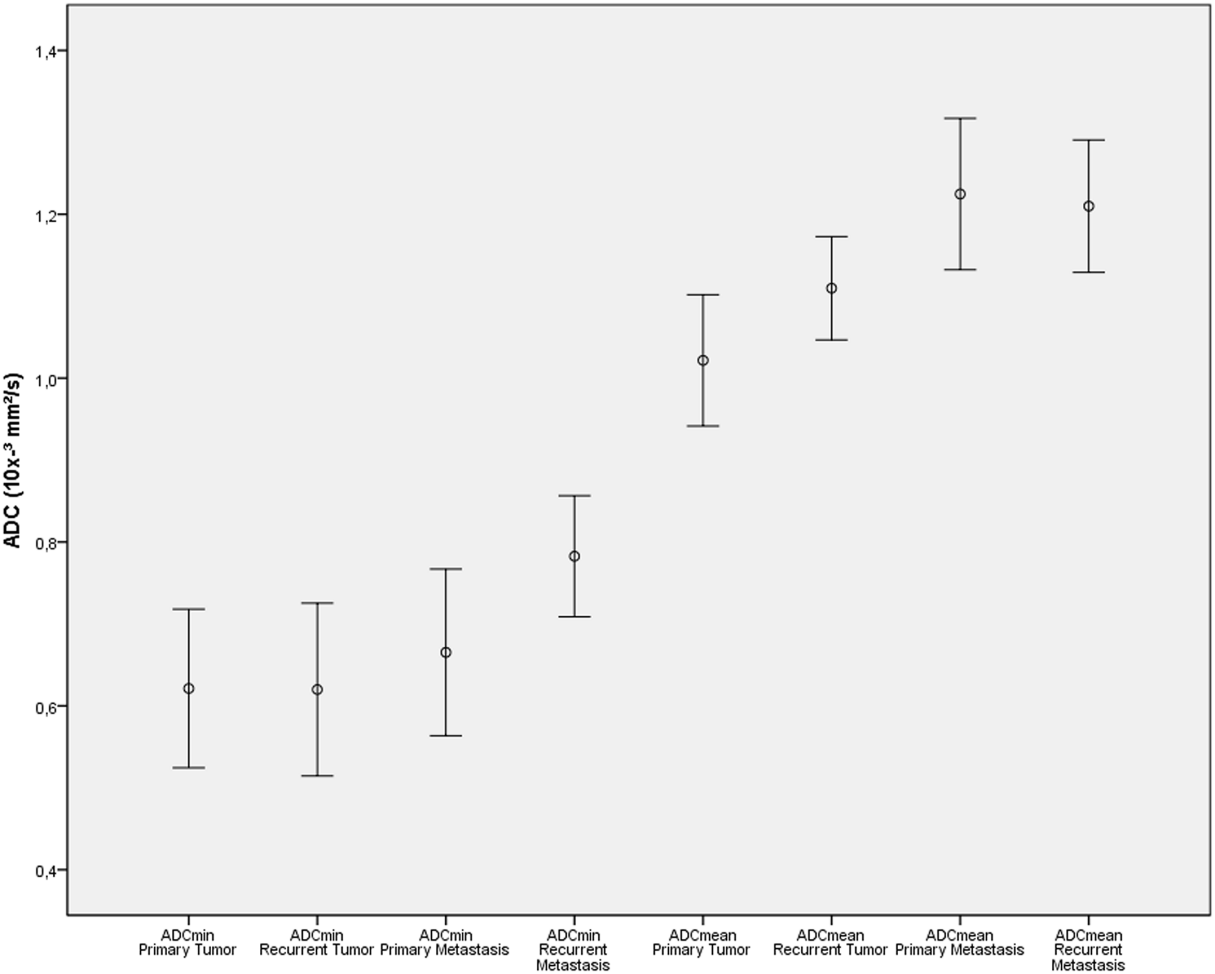
Absolute ADC values of primary and recurrent cervical cancer/metastasis presented through error bars.

All 31 patients successfully completed the whole-body PET/MRI examinations without any relevant side effects in an appropriate examination time of 30 minutes on average for whole body imaging. A total of 92 cancerous lesions were detected by simultaneous PET/MRI: primary tumors (n = 14), primary metastasis (n = 29 lymph nodes) recurrent tumors (n = 14) or recurrent metastasis (n = 35; 34 lymph nodes and 1 liver metastasis). Values for SUV and ADC (see S1 File) of these lesions are demonstrated in Figs 3 and 4 respectively. For primary tumors, mean values were 24.8±14.2 for SUV_max_ and 13.1±8.7 for SUV_mean_, for primary metastasis 13.5±6.6 for SUV_max_ and 8.0±4.3 for SUV_mean_, for recurrent tumors 17.4±5.7 for SUV_max_ and 10.0±3.5 for SUV_mean_ and for recurrent metastasis 14.5 ± 5.8 for SUV_max_ and 8.3±3.6 for SUV_mean_. There was a significant difference between SUVmax of primary tumors and SUVmax of recurrent tumors *(p<0*.*05)* but not between primary and recurrent metastases *(p = 0*.*39)*.

Assessment of ADC in primary tumors showed values of 0.62±0.17 x 10^−3^ mm/s² for ADC_min_ and 1.02±0.14 x 10^−3^ mm/s² for ADC_mean_. In primary metastases values amounted to 0.64±0.16 x 10^−3^ mm/s² for ADC_min_ and 1.22±0.24 x 10^−3^ mm/s² for ADC_mean_. Analysis of ADC parameters for recurrent tumors revealed average values of 0.62±0.18 x 10^−3^ mm/s² for ADC_min_ and 1.11±0.11 x 10^−3^ mm/s² for ADC_mean_ whereas recurrent metastatic disease showed values for ADC_min_ of 0.68±0.18 x 10^−3^ mm/s² and 1.15±0.23 x 10^−3^ mm/s² for ADC_mean_. Overall there was no significant difference between ADC values of primary or recurrent disease.

**Fig 5:**
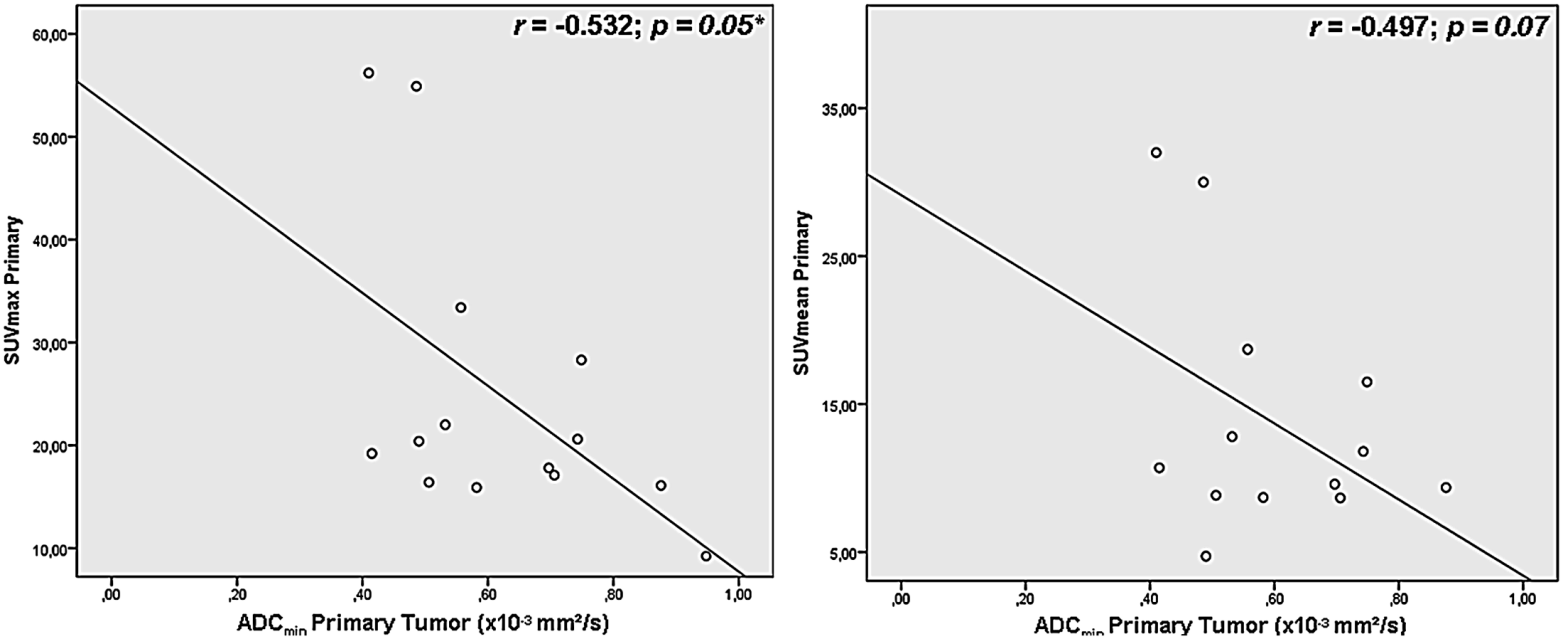
Correlation analysis of different SUV and ADC in primary tumors.

**Fig 6:**
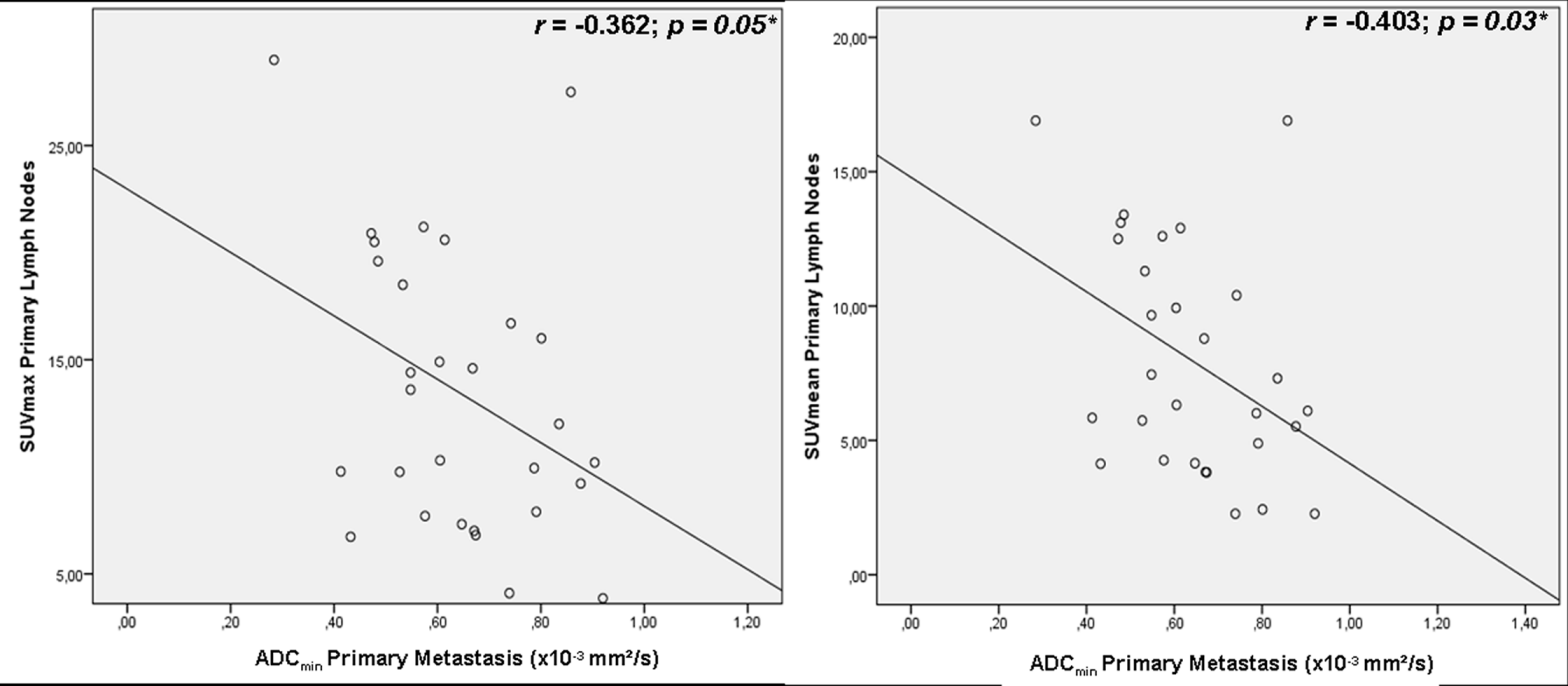
Correlation of SUV and ADC in primary metastasis.

Correlation analysis revealed a moderate significant inverse correlation in primary tumors (see Fig 5) for SUV_max_ versus ADC_min_ (r = -0.532, *p = 0*.*05*). The association between SUV_mean_ versus ADC_min_ (see Fig 5), SUV_mean_ versus ADC_mean_ and SUV_max_ versus ADC_mean_ missed the significance threshold and showed weak correlations (r = -0.497, *p = 0*.*07; r = -0*.*011*, *p = 0*.*09; r = 0*.*077*, *p = 0*.*79*). Primary metastasis showed weak inverse correlations for SUV_max_ and ADC_min_ (r = -0.362, *p = 0*.*05*, see Fig 6) and moderate correlations for SUV_mean_ and ADC_min_ (r = -0.403, *p = 0*.*03*, see Fig 6)–no inverse correlations were found for SUV_mean_ versus ADC_mean_ and SUV_max_ versus ADC_mean_ (r = 0.209 *p* = 0.28; r = 0.224, *p* = 0.243).

**Fig 7:**
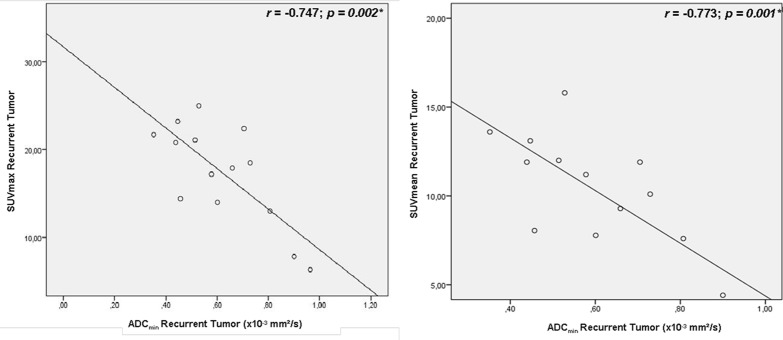
Correlation of SUV and ADC in recurrent tumor sites.

In recurrent local tumor sites we found strong correlations for SUV_max_ versus ADC_min_ (r = -0.747, *p = 0*.*002*, see Fig 7) and between SUV_mean_ and ADC_min_ (r = - 0.773, *p = 0*.*001*, see Fig 7). Weak, non—significant correlations were found for SUV_mean_ versus ADC_mean_ and SUV_max_ versus ADC_mean_ (r = -0.391 *p* = 0.16; r = -0.352 *p* = 0.22).

In recurrent metastases, SUV_max_ and SUV_mean_ showed no inverse correlation with ADC_min_ (r = -0.46, *p = 0*.*79* and r = + 0.81, *p = 0*.*65*, graphs not shown) nor with ADC_mean_ (r = 0.20 r = 0.229; *p* = 0.25 *p* = 0.19).

## Discussion

With the recent development of integrated PET/MRI scanners, new possibilities for quantitative molecular imaging have emerged. PET/MRI enables multimodal analysis of simultaneously acquired functional parameters which may contribute to a more sophisticated characterization of tumor biology and may also help to identify markers to predict response to therapy [18, 19].

Integration of DWI into diagnostic MR was initially used for stroke detection [20]. DWI hereby provides valuable information about the Brownian motion of water molecules (diffusion). Quantitative ADC values have been demonstrated to be negatively correlated with cellularity, yielding lower ADC values for various malignancies compared to surrounding healthy, inflammatory or scar tissue [21, 22]. In light of that, quantification and assessment of ADC might become a valuable tool for tumor delineation, tumor characterization and evaluation of therapy response [6, 9].

[^18^F-]FDG uptake correlates with the number of viable tumor cells and the resulting semi-quantitative read-out parameter SUV serves as a key player in oncologic imaging as it is the main non-invasive approach to quantify glucose metabolism. In numerous malignancies, ^18^F-FDG-PET is a useful tool to predict early response to therapy. A normalization of initial pathological ^18^F-FDG uptake and a significant SUV-decrease, respectively at an early stage of therapy is associated with an excellent prognosis and may help to identify patients who might benefit from de-escalation of antineoplastic therapy e.g. in lymphoma and non-small-lung cancer [23,24,25].

Both, ADC derived by DWI and SUV from [^18^ F-] FDG PET are described to be useful parameters for characterization of tumor lesions and for assessments of treatment response [26]. However, the benefit of using both parameters complementary in the diagnosis of malignant tumors and for response assessment remains unclear.

In our study, individual assessment of [^18^F]FDG SUVs showed significant differences between primary tumors and primary metastases. Kidd et al. [27] evaluated the prognostic significance of SUV_max_ in pelvic lymph node metastases in patients with cervical cancer and found similar results with higher values for primary cervical tumors (average SUV_max_ = 14) in comparison to primary pelvic lymph node metastases (average SUV_max_ = 6.9). Furthermore, SUV of pelvic lymph node metastases was found to be predictive for treatment response in the same study as well as pelvic recurrence risk and disease-specific survival in patients with cervical cancer during a mean follow-up time of 18 months.

Quantitative analysis of ADC has been performed in several studies for characterization and treatment response assessment in cervical cancer. Xue et al. [28] concluded that ADC values are helpful in assessing pathological subtypes and in differentiating cervical cancers by showing significantly different values for ADC_mean_ and ADC_min_ for adenocarcinomas, squamous cell carcinomas or poorly differentiated tumors. Another uni-modal MRI study dealing with the value of DWI in diagnosis of lymph node metastasis in patients with cervical cancer [29] showed a statistically significant difference in ADC_mean_ and ADC_min_ between metastatic and non-metastatic lymph nodes. In our study we could not find significant differences between ADC values of primary/recurrent tumors compared to primary/recurrent metastases.

Our study demonstrated an inverse correlation between SUVs and ADC values in primary and recurrent tumors. This main study finding in the largest cohort of cervical cancer patients so far imaged by integrated PET/MRI is supporting the theory of decreased ADC values which is indicating increased cellularity [9]. Similar findings [5] show that an increased FDG uptake also correlates with high cellularity of viable tumor cells and high tissue metabolism. Regarding the overall correlation of these two distinct parameters, existing literature describes inhomogeneous results for non-simultaneous image acquisition with PET/CT and standalone MRI for assessment of absolute SUV_max/mean_ and ADC_min/mean_ values [30, 13]. However, Ho et al. [30] demonstrated a significant inverse correlation between the rADC_min_, which is the relative ADC defined by ADC_min_/ADC_mean_, and SUV_max_ in primary cervical cancer with sequential PET/CT and MR. In a study performed by Nakajo et al. [31], 44 patients with breast cancer received a preoperative PET/CT and MRI (including DWI) within an average of 17 days between both. SUV_max_ and ADC (r = -0.486, p = 0.001) were significantly associated with histological grade, nodal status and vascular invasion. In another study by Mori et al. [32], a total of 104 patients with malignant pulmonary nodules were examined by PET/CT and MRI within a 2-week period showing an inverse correlation between SUV_max_ and ADC_min_. Contrary to these results, other data for breast tumor patients examined with PET/CT and MRI indicate only a weak, non-significant inverse association [33]. For head and neck tumors, Varoquaux et al. [13] described ADC and SUV as independent biomarkers. In contrast to these studies, in our study SUV and ADC were determined simultaneously with an integrated PET/MRI resulting in reduced registration artifacts due to an exact image fusion. Recently published data about the correlation of ADC and SUV in simultaneous PET/MRI imaging show significant inverse correlations between the ADC_mean_ and SUV_max_ in non-small cell lung cancer [34, 35] and an inverse correlation between SUV_max_ and ADC_min_ in a patient cohort of 19 women suffering from cervical carcinoma [14]. Compared to the latter study our results indicate almost similar correlations between the SUV and ADC of patients with cervical carcinomas and FDG-avid lymph nodes. This was even though ADC quantification is known to be susceptible to physical alterations depending on the choice of the b-values, which were slightly different in the above study [14]. Compared to the above-mentioned study of Ho et al. [30], where only the rSUV and rADC seemed to correlate inversely, we found a direct inverse correlation of SUV and ADC. Demonstrating an overall inverse correlation we found the highest correlation coefficients for SUV_max_ vs. ADC_min_ and SUV_mean_ vs. ADC_min_ for recurrent cervical cancer and weaker associations for primary cervical tumor sites. These findings might indicate differences within the genomic profile of primary and recurrent cervical cancer. As described by Martin et al. [36] and Hagemann et al. [37] genomic profiling and identification of different biomarkers are important to understand the pathogenesis of cervical cancer. Hagemann et al. tried to identify in both, lymph node micrometastases and recurrent cervical tumours up- or downregulated genes involved in several molecular pathways such as angiogenesis, oncogenic pathways, DNA repair mechanisms, migration, cell proliferation and apoptosis. The authors described, that in lymph node micrometastases most genes were downregulated or showed expressions equal to the levels found in matched primary cervical cancer. In recurrent cancer, almost all genes were upregulated at least two-fold compared to the expression profiles of primary cervical tumours, according to the authors possibly reflecting their aggressive biological behavior. Only two genes, the proapoptotic gene BAX and the tumour suppressor gene APC has been found to be consistently downregulated in lymph node metastases and recurrent cervical cancer [37]. Regarding our results, the stronger association of glucose metabolism and cellularity in recurrent cervical cancer compared to primary cervical cancer might also be influenced by different levels of gene expression in several molecular pathways.

As also described by Vanderhoek et al. [38], different SUV measures (SUVmax, SUVmean or SUVpeak) assess different tumor characteristics. SUVmax is measuring the tumor region of most intense metabolism, while SUVmean assesses overall metabolism in the tumor. As tumors tend to be heterogeneous, average metabolism and also average response to therapy of the entire tumor might be different from metabolism of one particular subregion within the tumor.

Assessment of tumor characteristics and treatment response using multiple SUV measures may offer a more complete tumor characterization. Furthermore, a combination of SUV measures might provide a more detailed tumor characterization.

SUVmean is described to have better reproducibility than SUVmax, which probably gives a less accurate result since SUVmax is only a single voxel-derived value. Regarding our results, the demonstrated significant inverse correlation between SUVmax and ADCmin in primary cervical cancer (without a significant association between SUVmean and ADCmin) might not be as robust as the significant inverse correlation of both, SUVmax and ADCmin and SUVmean and ADCmin in primary metastases.

This study has limitations that may have impacted the results. A direct histopathological or immunohistochemistry correlation was not performed but possibly would provide even deeper insights into tumor proliferation and thus the correlation of multifunctional PET/MR parameters. Another limitation is that only lesions were included that could be detected on both modalities. However, as we wanted to investigate a potential correlation of these parameters in a simultaneous hybrid system we needed to include only lesion visible in both modalities. Furthermore, due to performance of PET/CT prior to PET/MRI in our patient cohort, the average uptake time for FDG in PET/MRI is approximately 130 minutes, which could probably influence SUV measurements.

## Conclusion

The current study demonstrates the feasibility of a fast, reliable and simultaneous measurement of SUV and ADC values in an integrated PET/MRI. We found significant inverse correlations between SUV and ADC values in patients with primary or recurrent cervical cancer and metastases. The correlation of these parameters may contribute to a more sophisticated characterization of tumor biology in cervical cancer.

## Supporting Information

S1 File Datasheet containing Supporting Information for ADC and SUV. (XLS)
